# The impact of elevated dietary inflammatory potential on sarcopenic obesity: evidence from two observational studies

**DOI:** 10.3389/fnut.2025.1621199

**Published:** 2025-08-11

**Authors:** Xi Luo, Weiwei Jin, Shengcheng Mao

**Affiliations:** ^1^Department of Clinical Nutrition, Tongde Hospital of Zhejiang Province, Hangzhou, China; ^2^Department of Endocrinology, Tongde Hospital of Zhejiang Province, Hangzhou, China

**Keywords:** dietary inflammatory index, CRP-albumin-lymphocyte index, neutrophil-percentage-toalbumin ratio, sarcopenic obesity, NHANES

## Abstract

**Background and objective:**

Chronic low-grade inflammation plays a critical role in the onset and progression of both sarcopenia and obesity. Diet, as a well-known modifiable factor of low-grade inflammation, significantly impacts adverse health conditions, including obesity and sarcopenia. This study aims to explore the association between dietary inflammatory potential and sarcopenic obesity (SO).

**Methods:**

A total of 4,470 subjects from two National Health and Nutrition Examination Survey (NHANES) cycles (2015–2016 and 2017–2018) and 276 subjects enrolled at Tongde Hospital of Zhejiang Province between January 2024 and February 2025 were enrolled in the present study. Logistic regression was used to investigate the association between the dietary inflammatory index (DII) and SO. Moreover, the mediating effect of C-reactive protein-albumin-lymphocyte (CALLY) and neutrophil-percentage-to-albumin ratio (NPAR) was evaluated to investigate the association between DII and SO in the NHANES cohort.

**Results:**

In the NHANES cohort, logistic regression demonstrated a positive association between the DII score and SO (adjusted odds ratio (OR) _continuous_ = 1.19, 95% CI = 1.08, 1.32, *p* = 0.012; adjusted OR _tertile3vs1_ = 1.93, 95% CI = 1.28, 2.92, *p* for trend = 0.015). In the Chinese population cohort, a positive association also existed between DII and SO (adjusted OR _continuous_ = 1.59, 95% CI = 1.30, 1.94, *p* < 0.001; adjusted OR _tertile3vs1_ = 6.10, 95% CI = 2.72, 13.68, *p* for trend <0.001). Using the NHANES data, the mediation analysis indicated that CALLY mediated 39.49% of the association between DII and SO, while NPAR mediated 7.35%.

**Conclusion:**

An elevated DII score is positively associated with the risk of SO in adults. The association appeared to be partially mediated through inflammatory/nutritional pathways, suggesting that the DII score may serve as a valuable indicator for the identification of individuals at risk of SO.

## Introduction

Obesity, a multifaceted and non-communicable disorder, is rapidly developing into a global epidemic and has become a significant health concern in contemporary society (1). Earlier research has demonstrated that excessive body fat accumulation can induce an imbalanced production of various adipokines and promote the infiltration of macrophages and other immune cells in adipose tissue (AT) (2, 3). These activities lead to the release of a diverse array of pro-inflammatory cytokines and chemokines, resulting in the establishment of a chronic low-grade inflammation state both locally and systemically, referred to as “inflammaging” (2, 3). Key inflammatory molecules involved in inflammaging, such as tumor necrosis factor-alpha (TNF-α), interleukin-6 (IL-6), interleukin-1 (IL-1), and various chemokines, promote the infiltration of inflammatory cells that lead to muscle deterioration via activation of the NF-κB pathway (4). In AT, muscle progenitor cells may differentiate into an adipocyte-like phenotype due to paracrine signaling from cytokines (5–7). This differentiation leads to increased fatty infiltration and decreased muscular regeneration, thereby perpetuating a vicious cycle (5). The interaction between fatty infiltration and muscle loss could potentially initiate and exacerbate sarcopenic obesity (SO) (7–9), which may lead to more severe adverse health outcomes, such as reduced quality of life and increased risks of frailty, falls, disability, and even mortality (10, 11).

Many factors contribute to a low-grade inflammatory state, with diet being one of the most significant modifiable factors (12). Unhealthy dietary patterns are closely linked to elevated inflammatory markers (13, 14), resulting in increased adverse health outcomes, such as obesity, sarcopenia, and various chronic non-communicable diseases (15–20). The Dietary Inflammation Index (DII) is a scoring tool designed to assess the inflammatory potential of diets (21). A lower DII score reflects a more anti-inflammatory diet, whereas a higher DII score indicates a more pro-inflammatory diet.

Recently, the implications of dietary inflammatory potential for obesity and sarcopenia have garnered increasing attention (16, 22, 23). Numerous population-based studies have explored the correlation between the DII score and these conditions, highlighting a strong association between dietary inflammation and the risk of obesity and sarcopenia (16, 23–29). However, research exploring the relationship between DII and SO remains limited. Currently, SO is no longer exclusive to the elderly; lifestyle changes, including poor dietary habits and sedentary behaviors (SBs), have contributed to a trend of younger individuals being affected by pre-SO and SO (11). Therefore, gaining a deeper understanding of the relationship between the DII and SO would be more meaningful and could offer significant insights into developing effective dietary interventions for weight management.

The present study aimed to explore the association between SO and the inflammatory potential of daily diets in adults, using datasets from the National Health and Nutrition Examination Survey (NHANES) and Tongde Hospital of Zhejiang Province. Moreover, we explore whether inflammatory/nutritional indicators have a potential role in mediating the association between DII and SO using the NHANES dataset.

## Materials and methods

### Study population

**Figure 1:**
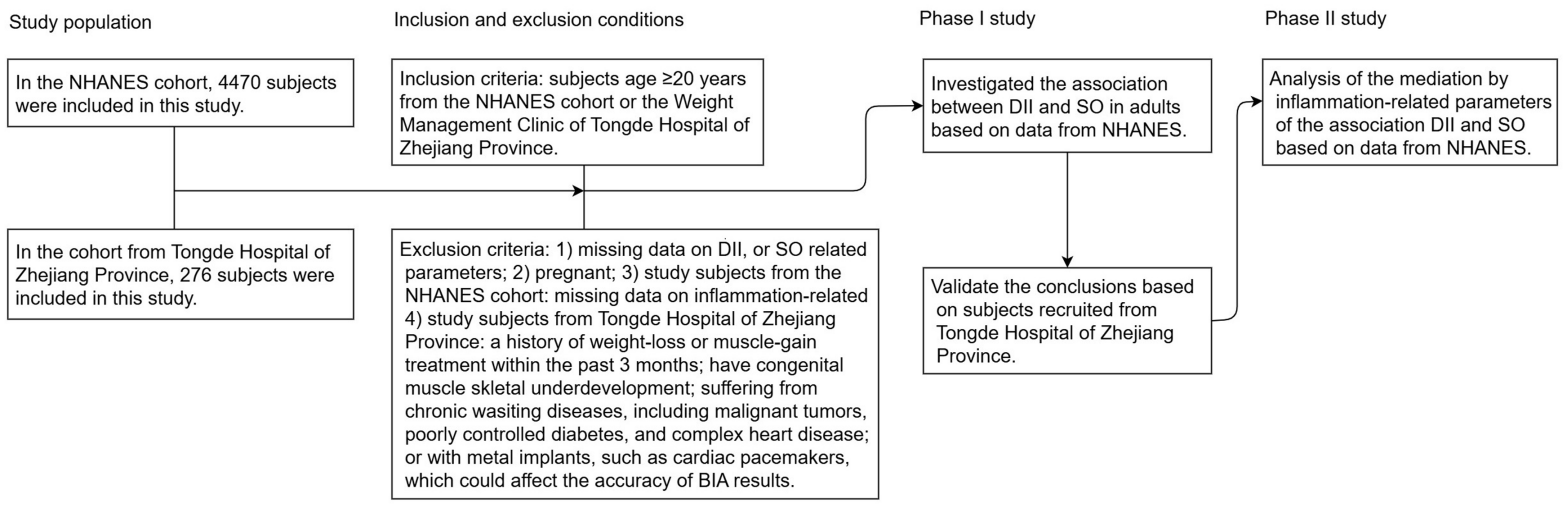
Flowchart of study participants. NHANES, National Health and Nutrition Examination Survey; DII, dietary inflammatory index; SO, sarcopenic obesity; BIA, bioelectrical impedance analysis.

The present study collected data from NHANES and Tongde Hospital in Zhejiang Province, and the detailed research process is illustrated in Figure 1. NHANES is a nationally representative, population-based cross-sectional survey conducted in the United States (U.S.). This survey includes demographic information, physical examination results, dietary details, and various health-related data. Comprehensive details about survey methodologies and data access can be found at https://www.cdc.gov/nchs/nhanes/about_nhanes.htm. The study protocol was approved by the NCHS Research Ethics Review Board, and all participants provided their written informed consent to participate in this study. The Chinese cohort enrolled patients who participated in the weight management program at Tongde Hospital of Zhejiang Province. All the researchers in this program received professional training. The study was approved by the Medical Ethics Committee of Tongde Hospital of Zhejiang Province (No. 2024-011-JY, No. 2024-012-JY), and all participants signed informed consent. All methods were performed in accordance with the relevant guidelines and regulations, and a full description is available online in the China Trial Register (ChiCTR2400080592, ChiCTR2400080393). The survey was performed in accordance with the relevant guidelines and regulations.

In the U.S. population cohort, the study utilized datasets from two NHANES cycles (2015–2016 and 2017–2018). To ensure the integrity and reliability of the study’s results, we implemented specific exclusion criteria, which included individuals <20 years of age (*n* = 7,937), pregnant (*n* = 94), and those missing data on DII score (*n* = 1,525), SO-related parameters (*n* = 4,964), and inflammation/nutritional parameters (*N* = 235). After applying these criteria, the study ultimately included a cohort of 4,470 subjects, as detailed in Supplementary Figure 1. In the Chinese cohort, a total of 304 patients who participated in the weight management program at Tongde Hospital in Zhejiang Province between January 2024 and February 2025 were enrolled. All subjects were classified as overweight or obese based on their body mass index (BMI) and met the following exclusion criteria: individuals (I) aged <20 years; (II) pregnant; (III) a history of weight-loss or muscle-gain treatment within the past 3 months; (IV) congenital muscle or skeletal underdevelopment; (V) suffering from chronic wasting diseases, including malignant tumors, poorly controlled diabetes, and complex heart disease; and (VI) presence of metal implants, such as cardiac pacemakers, which could affect the accuracy of bioelectrical impedance analysis (BIA) results. Twenty-eight subjects were excluded due to missing dietary intake data, body composition, or covariates, resulting in a cohort of 276 subjects (Supplementary Figure 2).

### Assessment of dietary intake

In the NHANES cohort, all participants are eligible for two 24-h dietary recall interviews. The first dietary recall interview is collected in person in the Mobile Examination Center (MEC), and the second interview is collected by telephone 3–10 days later. To estimate participants’ nutrient intake data, the present study calculated the mean values from two reliable 24-h recall interviews, specifically excluding supplements and medications (20). This data was subsequently used to calculate the DII.

In the Chinese population cohort, unannounced dietary intake information was evaluated using three reliable 24-h recall interviews, comprising two weekdays and one weekend day. All participants engaged in face-to-face interviews, utilizing food models and pictures to improve the accuracy of the dietary intake data. The participants’ nutrient intake data were calculated using a computerized app in accordance with the China Food Composition (National Institute of Nutrition and Food Safety, China CDC) (26). The mean nutrient intake data were subsequently used to calculate the DII.

### Dietary inflammation index

The DII is a scoring system developed by Shivappa et al., designed to assess the inflammatory potential of dietary intakes (21). Initially, it comprised 45 dietary items and nutrients. Importantly, the DII score maintains its validity even if no more than 30 of these dietary items and nutrients are utilized (21). In the NHANES cohort, we calculated the DII score based on 27 dietary items and nutrients, which include protein, carbohydrate, total fat, n-3 fatty acids, n-6 fatty acids, polyunsaturated fatty acids (PUFAs), monounsaturated fatty acids (MUFAs), saturated fat, cholesterol, dietary fiber, vitamin A, β-carotene, thiamin, riboflavin, vitamin B_6_, vitamin B_12_, folic acid, niacin, vitamin C, vitamin D, vitamin E, iron, zinc, magnesium, selenium, caffeine, and alcohol (30). In the China population cohort, 27 nutrients were used for the calculation of the DII score, which includes energy, protein, carbohydrate, total fat, PUFAs, MUFAs, saturated fat, cholesterol, dietary fiber, vitamin A, β-carotene, thiamin, riboflavin, vitamin B_6_, vitamin B_12_, folic acid, niacin, vitamin C, vitamin D, vitamin E, iron, zinc, magnesium, selenium, isoflavones, caffeine, and alcohol. The DII score was calculated by the following equation (21):


$$\mathrm{DII}=(\begin{array}{l} Z\;\text{score}\times \text{the inflammatory effect}\\ \text{score of each dietary component} \end{array});$$

$$\begin{array}{l} Z\;\text{score}=(\text{daily mean intake}-\text{global daily mean intake})\\ /\text{standard deviation}; \end{array}$$

Zscore=Zscore→(converted toapercentile score)×2−1.

### Assessment of SO

In the NHANES cohort, dual-energy X-ray absorptiometry (DXA) was used to measure appendicular skeletal muscle mass (ASM). The sarcopenia index (SI) was calculated by the following formula: SI = ASM (kg)/BMI (kg/m^2^). Sarcopenia was defined using the sex-specific SI cutoffs established by the Foundation for the National Institutes of Health (FNIH), which are <0.789 for males and <0.512 for females (31). BMI was calculated using the equation: the ratio of weight (kg)/height (m) squared. It was categorized as overweight (25.0 kg/m^2^ ≤ BMI < 30.0 kg/m^2^) and obesity (BMI ≥ 30.0 kg/m^2^) according to the WHO reference. Abdominal obesity was defined using waist circumference (WC) cutoffs, which are ≥102 cm for males and ≥88 cm for females. In the Chinese cohort, we assessed body composition with a multi-frequency BIA analyzer (InBody720, Biospace, Korea). Body composition, including body weight (BW), ASM, fat mass (FM), and percent body fat (PBF). Height was measured on a height-weight scale (HCM-800, SHENGYUAN, China). WC was measured in a standing position, at the midpoint between the anterior superior iliac spine and the lower rib, after normal expiration, using a non-stretchable tape. The appendicular skeletal muscle mass index (ASMI) was calculated by the following formula: ASMI (kg/m^2^) = ASM (kg)/Height^2^ (m^2^). Sarcopenia was defined using the sex-specific ASMI cutoffs (via BIA) established by the Asian Working Group for Sarcopenia (AWGS) 2019 Consensus, which are <7.0 kg/m^2^ for males and <5.7 kg/m^2^ for females (32). BMI was categorized as overweight (24.0 kg/m^2^ ≤ BMI < 28.0 kg/m^2^) and obesity (BMI ≥ 28.0 kg/m^2^) according to the Chinese BMI cutoff values. Abdominal obesity was defined using Chinese WC cutoffs, which are ≥90 cm for males and ≥85 cm for females. In the present study, SO was defined as the coexistence of sarcopenia and obesity (overweight and obesity (OAO) or abdominal obesity) (17).

### Assessment of inflammatory/nutritional indicators

Following the NHANES cohort, laboratory parameters, including albumin, C-reactive protein (CRP), and complete blood count (CBC) (lymphocytes, neutrophils, monocytes, and platelets) were collected. The inflammatory/nutritional indicators, including CRP-albumin-lymphocyte (CALLY) index and neutrophil-percentage-to-albumin ratio (NPAR), were calculated by the following formulas: CALLY = albumin × lymphocyte count /CRP (33); NPAR = neutrophil percentage (in total WBC count) × 100/albumin (34).

### Assessment of covariates

In the NHANES cohort, we obtained the particular methodologies and caliber of determination for every covariate control approach from NHANES.^1^ The covariates of the present study include age, gender, ethnicity, marital status, education level, family poverty-to-income ratio (PIR), smoking status, physical activity (PA) level, diabetes mellitus (DM), hypertension, and stroke history. Smoking status was categorized into never (smoked <100 cigarettes in life), former (smoked ≥100 cigarettes in life and does not smoke cigarettes now), and now (smoked ≥100 cigarettes in life and still smokes cigarettes now). Drinking status was categorized into mild (≤2 alcohol drinks/day for males or ≤1 alcohol drink/day for females on average over the past 1 year), moderate (2–4 alcohol drinks/day for males or 1–3 alcohol drink/day for females on average over the past 1 year), and heavy (≥5 alcohol drinks/day for males or ≥4 alcohol drink/day for females on average over the past 1 year) drinkers. PA levels were categorized into low PA (<500 MET/week) and high PA (≥500 MET/week) (35). Hypertension was diagnosed with a self-reported history of hypertension, systolic blood pressure (BP) ≥ 140 mmHg, and/or diastolic BP ≥ 90 mmHg. DM was indicated by any of the following criteria: a self-reported history of DM, use of insulin or oral antidiabetic medications, fasting glucose ≥126 mg/dL, 2-h glucose (OGTT) ≥ 200 mg/dL, or glycosylated hemoglobin (HbAlc) ≥ 6.5%. Stroke was identified through self-reported previous diagnosis.

In the Chinese population cohort, the covariates include age, gender, marital status, education level, smoking status, PA level, DM, hypertension, and stroke history. WC was measured in a standing position, at the midpoint between the anterior superior iliac spine and the lower rib, after normal expiration, using a non-stretchable tape. PA levels were assessed using the International Physical Activity Questionnaire (IPAQ) (36). The short-format IPAQ was applied to record the total time participants spent in sedentary, walking, moderate, and vigorous activities per week. The PA levels were calculated and categorized into low PA (<500 MET/week) and high PA (≥500 MET/week). Data on hypertension, hyperlipidemia, and stroke prevalence were collected through self-reported surveys.

### Statistical analysis

All analyses were conducted using R version 4.4.0 (The R Foundation),^2^ along with the Storm Statistical Platform^3^ and EmpowerStats (X&Y Solutions, Inc., Boston, MA, USA)^4^. A *p*-value of <0.05 was considered statistically significant. We conducted a comparison of baseline characteristics between the SO and non-SO groups. For NHANES study participants, continuous variables were expressed as mean (standard error, SE), while categorical variables were presented as number (n) (percentage, %). Due to the intricate sampling methodology and sample weight, NHANES provided nationally representative data. Accordingly, we utilized NHANES sample weights spanning 2015–2018, derived from day-2 dietary sample weights. For the Chinese cohort, continuous variables were expressed as mean (standard deviation, SD), and categorical variables were presented as *n* (%).

In the first part of the present study, we investigated the association between DII and SO based on datasets from NHANES and Tongde Hospital in Zhejiang Province. For NHANES study participants: (1) We constructed linear regression and logistic regression analyses with univariate and multivariate models. Model 1 was a non-adjusted model. Model 2 was adjusted for age, gender, ethnicity, marital status, education level, PIR, smoking status, and PA level. Model 3 was adjusted for age, gender, ethnicity, marital status, education level, PIR, smoking status, PA level, DM, hypertension, and stroke history. (2) We assessed the linear relationship between DII and risk of SO by restricted cubic spline (RCS) regression. (3) We implemented both sensitivity and subgroup analyses to strengthen the reliability of our data analysis. First, we used multiple imputation techniques to address missing values in variables such as marital status (0.02%), education level (0.02%), PIR (8.64%), smoking status (0.04%), DM (1.32%), hypertension (0.09%), and stroke (0.04%). The findings indicated no significant differences between the datasets before and after imputation. Then, we performed stratified and interaction analyses across various parameters, including age, gender, ethnicity, marital status, education level, smoking status, PA level, and histories of DM, hypertension, and stroke. Subsequently, we used linear regression and logistic regression to validate our findings with data obtained from subjects recruited at Tongde Hospital in Zhejiang Province and applied RCS regression to further explore the association between DII and SO. In the second part of the present study, we investigated the mediation by inflammatory/nutritional indicators of the association between DII and SO in the NHANES cohort.

## Results

### Baseline characteristics of the study population

A total of 4,470 subjects from NHANES and 276 subjects from Tongde Hospital of Zhejiang Province were enrolled in the present study. In the NHANES cohort, 49.00% were males, and the mean age was 39.18 (SE: 0.30) years. The mean DII score was 0.73 (SE: 0.07), ranging from −5.29 to 4.96. Among the 4,470 participants, 8.00% were diagnosed with SO. The mean SI was 0.81 (SE: 0.01), with SIs of 0.97 (SE: 0.01) among males and 0.66 (SE: 0.01) among females. The comparison of baseline characteristics between the non-SO and SO groups is shown in Table 1. Significant differences were observed between the two groups in terms of DII score, gender, age, ethnicity, education level, drinking status, PA level, BMI, WC, waist-to-height ratio (WHtR), SI, SVR, PIR, lymphocytes, neutrophils, CRP, and histories of DM, hypertension, and stroke (all *p* < 0.05). There was no significant difference found in marital status, smoking status, monocytes, and platelets (all *p* > 0.05).

**Table 1:** Characteristics of participants from the NHANES.

Variables	Non-SO *n*^a^ = 4,026	SO *n*^a^ = 444	Statistic	*p*
Gender, *n*^a^ (%)			*χ*^2^ = 15.47	**0.004**
Male	1,906 (48.13)	244 (58.97)		
Female	2,120 (51.87)	200 (41.03)		
Age, years, Mean (SE)	38.84 (0.34)	43.13 (1.00)	*t* = 3.66	**<0.001**
BMI, kg/m^2^, Mean (SE)	28.33 (0.17)	35.77 (0.41)	*t* = 17.48	**<0.001**
WC, cm, Mean (SE)	96.27 (0.43)	112.70 (0.90)	*t* = 17.38	**<0.001**
WHtR, Mean (SE)	0.57 (0.00)	0.70 (0.01)	*t* = 24.02	**<0.001**
SI, kg/m^2^, Mean (SE)	0.83 (0.00)	0.64 (0.01)	*t* = −18.87	**<0.001**
SVR, kg/cm^2^, Mean (SE)	0.33 (0.01)	0.17 (0.01)	*t* = −14.04	**<0.001**
PIR, Mean (SE)	3.02 (0.06)	2.32 (0.11)	*t* = −6.36	**<0.001**
Ethnicity, *n*^a^ (%)			*χ*^2^ = 169.50	**<0.001**
Mexican American	603 (10.14)	181 (30.08)		
Other Hispanic	462 (7.82)	77 (14.88)		
Non-Hispanic White	1,288 (58.84)	95 (41.68)		
Non-Hispanic Black	865 (11.69)	23 (3.27)		
Other ethnicity-including multi-racial	808 (11.51)	68 (10.09)		
Education level, *n*^a^ (%)			*χ*^2^ = 107.50	**<0.001**
Less than 9th grade	231 (3.18)	84 (11.24)		
9–11th grade	411 (7.03)	67 (11.24)		
High School or equivalent	891 (22.87)	117 (31.79)		
Some college or AA degree	1,363 (33.82)	109 (29.96)		
College graduate or above	1,129 (33.11)	67 (15.78)		
Marital status, *n*^a^ (%)			*χ*^2^ = 17.24	0.235
Married	1,940 (49.21)	242 (48.68)		
Widowed	57 (1.32)	6 (1.57)		
Divorced	341 (8.48)	44 (11.11)		
Separated	145 (2.80)	19 (5.91)		
Never married	1,041 (25.91)	82 (20.37)		
Living with partner	501 (12.29)	51 (12.36)		
Smoking, *n*^a^ (%)			*χ*^2^ = 1.87	0.606
Never	2,498 (60.01)	277 (56.34)		
Former	668 (19.70)	84 (21.22)		
Now	858 (20.29)	83 (22.44)		
Drinking, *n*^a^ (%)			*χ*^2^ = 20.94	**0.014**
Mild	1,316 (43.12)	103 (31.28)		
Moderate	1,079 (38.81)	99 (42.68)		
Heavy	551 (18.08)	70 (26.05)		
PA level, *n*^a^ (%)			*χ*^2^ = 35.68	**<0.001**
Low PA	1,127 (23.81)	187 (38.06)		
High PA	2,899 (76.19)	257 (61.94)		
Hypertension, *n*^a^ (%)	1,089 (24.44)	175 (41.61)	*χ*^2^ = 50.52	**<0.001**
DM, *n*^a^ (%)	429 (8.36)	106 (23.63)	*χ*^2^ = 88.72	**<0.001**
Stroke, *n*^a^ (%)	63 (1.15)	14 (3.73)	*χ*^2^ = 16.24	**0.009**
Lymphocytes (1,000 cells/uL), Mean (SE)	2.29 (0.02)	2.42 (0.05)	*t* = 2.38	**0.024**
Neutrophils (1,000 cell/uL), Mean (SE)	4.30 (0.05)	4.93 (0.13)	*t* = 4.19	**<0.001**
Monocytes (1,000 cells/uL) Mean (SE)	0.58 (0.01)	0.61 (0.01)	*t* = 1.65	0.110
Platelets, (1,000 cells/uL), Mean (SE)	244.85 (1.43)	253.81 (6.40)	*t* = 1.48	0.149
CRP, (mg/L), Mean (SE)	3.34 (0.15)	6.60 (0.59)	*t* = 5.51	**<0.001**
CALLY, Mean (SE)	1,666.02 (98.07)	419.86 (26.54)	*t* = −12.14	**<0.001**
NPAR, Mean (SE)	13.31 (0.07)	14.36 (0.23)	*t* = 4.32	**<0.001**
DII, Mean (SE)	0.68 (0.07)	1.28 (0.14)	*t* = 4.16	**<0.001**

a Unweighted number of observations in dataset.

SE, standard error; *t*, t-test, *χ*^2^, chi-square test; NHANES, National Health and Nutrition Examination Survey; SO, sarcopenic obesity; BMI, body mass index; WC, waist circumference; WHtR, waist-to-height ratio; SI, sarcopenia index; SVR, skeletal muscle mass-to-visceral area ratio; family poverty-to-income ratio; PA, physical activity; DM, diabetes mellitus; CALLY, C-reactive protein-albumin-lymphocyte; NPAR, neutrophil-percentage-to-albumin ratio; DII, dietary inflammation index.*P*-value shown in bold indicate statistical significance (*P* < 0.05).

In the Chinese cohort, 51.81% were males, and the mean age was 42.98 (SD: 12.34) years. The mean DII score was 0.84 (SD: 1.61), ranging from −3.50 to 4.65. Among the 276 OAO participants, 36.53% were diagnosed with sarcopenia. The mean ASMI was 7.23 (SD: 1.43) kg/m^2^. ASMIs were 7.90 (SD: 1.27) among males and 6.51 (SD: 1.22) among females. The comparison of baseline characteristics between the non-SO and SO groups is shown in Table 2. Significant differences were observed between the two groups in terms of DII score, gender, age, marital status, BMI, WC, WHtR, PBF, ASMI, and history of DM (all *p* < 0.05). There was no significant difference found in education level, smoking status, drinking status, PA level, DM, and stroke (all *p* > 0.05).

**Table 2:** Characteristics of participants from the Tongde Hospital of Zhejiang Province.

Variables	Non-SO *n* = 177	SO *n* = 99	Statistic	*p*-value
Gender, *n* (%)			*χ*^2^ = 9.53	**0.002**
Male	104 (58.76)	39 (39.39)		
Female	73 (41.24)	60 (60.61)		
Age, years, Mean (SD)	40.45 ± 11.89	47.51 ± 12.31	*t* = −4.67	**<0.001**
BMI, kg/m^2^, Mean (SD)	28.61 ± 5.18	24.93 ± 0.72	*t* = 9.30	**<0.001**
WC, cm, Mean (SD)	90.80 ± 10.35	82.46 ± 3.75	*t* = 9.64	**<0.001**
WHtR, Mean (SD)	0.55 ± 0.06	0.53 ± 0.03	*t* = 2.93	**0.004**
PBF, %, Mean (SD)	32.96 ± 7.43	37.42 ± 4.34	*t* = −6.29	**<0.001**
ASMI, kg/m^2^, Mean (SD)	7.91 ± 1.30	6.02 ± 0.61	*t* = 16.35	**<0.001**
SVR, kg/cm^2^, Mean (SE)	0.26 ± 0.15	0.22 ± 0.19	*t* = 1.88	0.061
Education level, *n* (%)			*χ*^2^ = 3.84	0.050
High School or below	56 (31.64)	43 (43.43)		
College or above	121 (68.36)	56 (56.57)		
Marital status, *n* (%)			*χ*^2^ = 5.90	**0.015**
Couple	134 (75.71)	87 (87.88)		
Single or separated	43 (24.29)	12 (12.12)		
Smoking status, *n* (%)			*χ*^2^ = 0.02	0.889
Yes	10 (5.65)	6 (6.06)		
No	167 (94.35)	93 (93.94)		
Drinking status, *n* (%)			*χ*^2^ = 0.96	0.327
Yes	45 (25.42)	20 (20.20)		
No	132 (74.58)	79 (79.80)		
PA level, *n* (%)			*χ*^2^ = 3.56	0.059
Low PA	133 (75.14)	84 (84.85)		
High PA	44 (24.86)	15 (15.15)		
Hypertension, *n* (%)	15 (8.52)	2 (2.02)	*χ*^2^ = 4.62	**0.032**
DM, *n* (%)	6 (3.39)	5 (5.05)	*χ*^2^ = 0.13	0.722
Stroke, *n* (%)	1 (0.56)	0 (0.00)	–	1.000
DII, Mean (SD)	0.50 ± 1.60	1.45 ± 1.44	*t* = −5.05	**<0.001**

SD, standard deviation; *t*, t-test; *χ*^2^, chi-square test; SO, sarcopenic obesity; BMI, body mass index; WC, waist circumference; WHtR, waist-to-height ratio; PBF, percent body fat; ASM, appendicular skeletal muscle mass; ASMI, appendicular skeletal muscle mass index; SVR, skeletal muscle mass to visceral area ratio; PA, physical activity; DM, diabetes mellitus; DII, dietary inflammation index.*P*-value shown in bold indicate statistical significance (*P* < 0.05).

### Associations of DII with body composition index

In the NHANES cohort, the associations of the DII with body composition index were evaluated. First, we explored the relationship between the DII and body composition index in U.S. adults. Our findings revealed a negative association of DII with SI (Model 1: *β* = −2.11, 95% CI = −2.48, −1.74, *p* < 0.001; Model 2: *β* = −3.15, 95% CI = −4.02, −2.28, *p* < 0.001; Model 3: *β* = −3.06, 95% CI = −3.95, −2.16, *p* < 0.001) and SVR (Model 1: *β* = −1.24, 95% CI = −1.64, −0.85, *p* < 0.001; Model 2: *β* = −1.62, 95% CI = −2.20, −1.03, *p* < 0.001; Model 3: *β* = −1.63, 95% CI = −2.24, −1.03, *p* < 0.001) in the unadjusted and multivariable-adjusted models (Table 3). In contrast, there was a positive association of DII with BMI (Model 1: *β* = 0.04, 95% CI = 0.03, 0.05, *p* < 0.001; Model 2: *β* = 0.03, 95% CI = 0.02, 0.04, *p* < 0.001; Model 3: *β* = 0.03, 95% CI = 0.02, 0.04, *p* < 0.001), WC (Model 1: *β* = 3.14, 95% CI = 2.39, 3.89, *p* < 0.001; Model 2: *β* = 2.53, 95% CI = 1.70, 3.37, *p* < 0.001; Model 3: *β* = 2.46, 95% CI = 1.59, 3.34, *p* < 0.001), and WHtR (Model 1: *β* = 0.01, 95% CI = 0.01, 0.02, *p* < 0.001; Model 2: *β* = 0.01, 95% CI = 0.01, 0.02, *p* < 0.001; Model 3: *β* = 0.01, 95% CI = 0.01, 0.02, *p* < 0.001) in the unadjusted and multivariable-adjusted models (Table 3). In the study subjects recruited from Tongde Hospital of Zhejiang Province, there also existed a negative association between DII with ASMI and SVR, and a positive association between DII and WHtR and PBF in the unadjusted and multivariable-adjusted models (Table 3).

**Table 3:** Association between DII and body composition index.

Variables	Model 1 *β* (95% CI)	*p*	Model 2 *β* (95% CI)	*p*	Model 3 *β* (95% CI)	*p*
The NHANES cohort						
SI	−2.11 (−2.48, −1.74)	**<0.001**	−3.15 (−4.02, −2.28)	<0.001	−3.06 (−3.95, −2.16)	<0.001
SVR	−1.24 (−1.64, −0.85)	**<0.001**	−1.62 (−2.20, −1.03)	<0.001	−1.63 (−2.24, −1.03)	<0.001
BMI	0.04 (0.03, 0.05)	**<0.001**	0.03 (0.02, 0.04)	<0.001	0.03 (0.02, 0.04)	0.001
WC	3.14 (2.39, 3.89)	**<0.001**	2.53 (1.70, 3.37)	<0.001	2.46 (1.59, 3.34)	<0.001
WHtR	0.01 (0.01, 0.02)	**<0.001**	0.01 (0.01, 0.02)	<0.001	0.01 (0.01, 0.02)	<0.001
The Chinese cohort						
ASMI	−0.19 (−0.30, −0.09)	**<0.001**	−0.14 (−0.22, −0.05)	0.002	−0.16 (−0.24, −0.08)	<0.001
SVR	−0.03 (−0.04, −0.01)	**<0.001**	−0.02 (−0.03, −0.01)	<0.001	−0.02 (−0.03, −0.01)	<0.001
BMI	0.08 (−0.25, 0.41)	0.643	0.06 (−0.25, 0.38)	0.685	0.03 (−0.28, 0.33)	0.851
WC	−0.02 (−0.72, 0.67)	0.945	0.22 (−0.43, 0.87)	0.503	0.05 (−0.57, 0.872)	0.872
WHtR	0.01 (0.01, 0.01)	**0.003**	0.01 (0.01, 0.01)	0.005	0.01 (0.01, 0.01)	0.016
PBF	1.44 (0.97, 1.91)	**<0.001**	1.05 (0.70, 1.40)	<0.001	1.05 (0.70, 1.40)	<0.001

In the NHANES cohort, Model 1, non-adjusted model. Model 2, adjusted for age, gender, ethnicity, marital status, education level, PIR, smoking status, and PA level. Model 3, adjusted for age, gender, ethnicity, marital status, education level, PIR, smoking status, PA level, DM, hypertension, and stroke history. In the Chinese cohort, Model 1, non-adjusted model. Model 2, adjusted for age, gender, marital status, education level, and PA level. Model 3, adjusted for age, gender, marital status, education level, PA level, DM, hypertension, and stroke history. NHANES, National Health and Nutrition Examination Survey; DII, dietary inflammatory index; PIR, family poverty-to-income ratio; PA, physical activity; BMI, body mass index; WC, waist circumference; WHtR, waist to height ratio; PBF, percent body fat; SI, sarcopenia index; ASMI, appendicular skeletal muscle mass index; SVR, skeletal muscle mass to visceral area ratio; DM, diabetes mellitus; CI, confidence interval.

### Association between DII and SO

**Figure 2:**
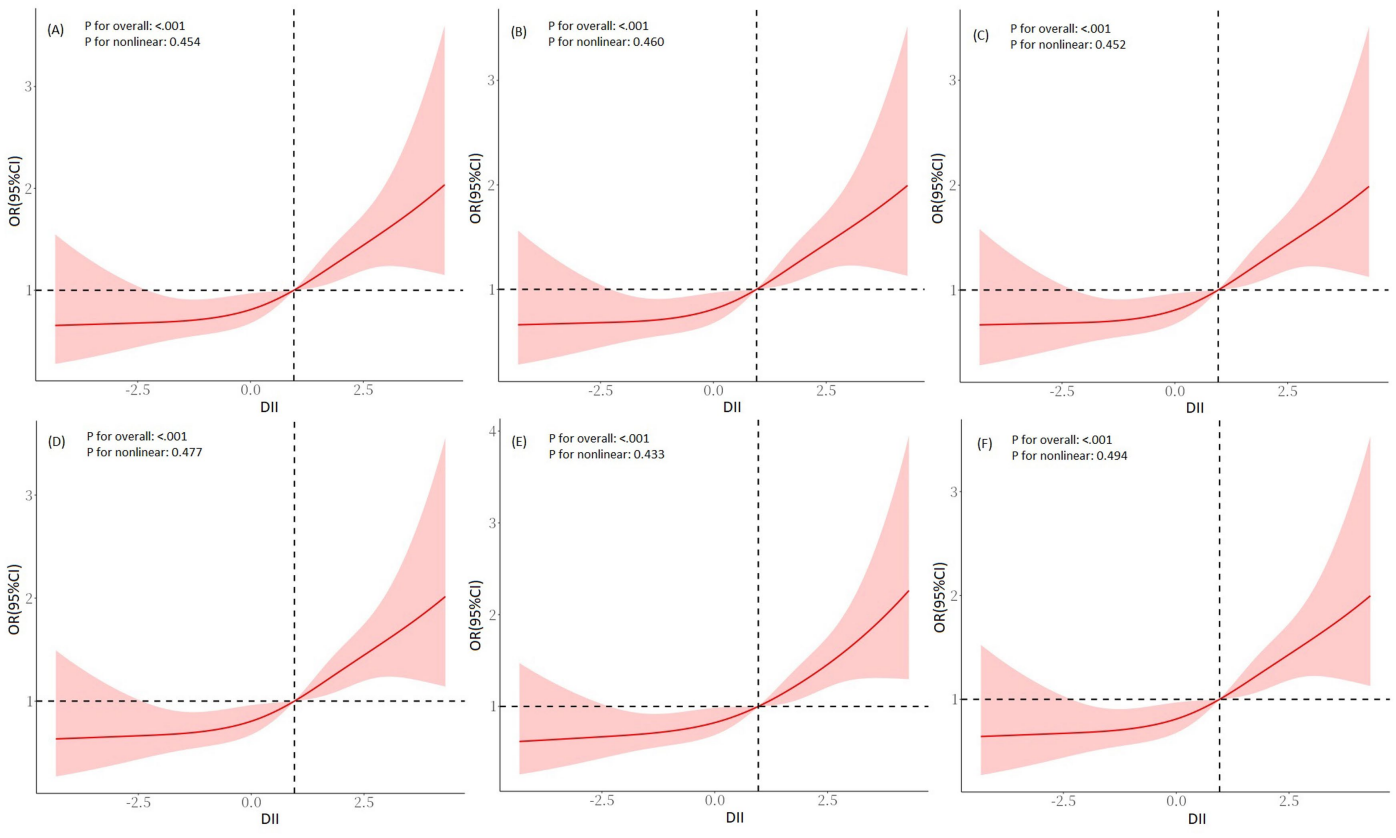
RCS regression analysis for DII and SO in the NHANES cohort. **(A)** Pre-imputation, **(B)** imputation 1, **(C)** imputation 2, **(D)** imputation 3, **(E)** imputation 4, and **(F)** imputation 5. Adjusted for age, gender, ethnicity, marital status, education level, PIR, smoking status, PA level, DM, hypertension, and stroke history. RCS, restricted cubic spline; DII, dietary inflammatory index; SO, sarcopenic obesity; PIR, family poverty-to-income ratio; PA, physical activity; DM, diabetes mellitus.

In the NHANES cohort, we explored the relationship between DII and SO. In Model 1 (crude model), a statistically significant association between increased odds ratios (ORs) for SO and DII scores was observed, with an OR of 1.19 (95% CI = 1.09, 1.30, *p* < 0.001, Table 4). The OR in model 2 was 1.21 (95% CI = 1.10, 1.34, *p* = 0.003, Table 4) after it was adjusted for age, gender, ethnicity, marital status, education level, PIR, smoking status, and PA status, while in Model 3 it was 1.19 (95% CI = 1.08, 1.32, *p* = 0.012, Table 4) after it was additionally adjusted for history of DM, hypertension, and stroke. We also converted DII into tertiles, and categorical DII displayed a significant relationship with SO (Model 1: OR _tertile3vs1_ = 1.95, 95% CI = 1.41, 2.70, *p* for trend <0.001, Table 4). After complete adjustment, categorical DII still showed a significant correlation with SO (Model 3: OR _tertile3vs1_ = 1.93, 95% CI = 1.28, 2.92, *p* for trend = 0.015, Table 4). In the RCS regression analysis (Figure 2A), we found that the association between DII and the risk of SO was monotonically increasing. To ensure the robustness of our findings, we examined the association between DII and SO using imputed data. The analysis confirmed that there were no qualitative changes in the results, as detailed in Figure 2. Subsequently, we used age, gender, ethnicity, marital status, education level, smoking status, PA level, BMI, abdominal obesity, DM, hypertension, and stroke history as stratification variables. We performed stratified analysis to evaluate the association between DII and SO in stratified populations. As shown in Supplementary Table 1, the above factors were not interactive factors in this study (all *p*-values for interaction >0.05).

**Table 4:** Association between DII and SO.

Variables	The NHANES cohort			The Chinese cohort		
	Model 1 OR (95% CI), *p*	Model 2 OR (95% CI), *p*	Model 3 OR (95% CI), *p*	Model 1 OR (95% CI), *p*	Model 2 OR (95% CI), *p*	Model 3 OR (95% CI), *p*
DII continuous	1.19 (1.09, 1.30), **<0.001**	1.21 (1.10, 1.34), **0.003**	1.19 (1.08, 1.32), **0.012**	1.51 (1.26, 1.80), **<0.001**	1.53 (1.26, 1.86), **<0.001**	1.59 (1.30, 1.94), **<0.001**
DII tertiles						
T1	Ref.	Ref.	Ref.	Ref.	Ref.	Ref.
T2	1.09 (0.74, 1.62), 0.670	1.12 (0.69, 1.81), 0.658	1.06 (0.65, 1.73), 0.825	7.59 (3.59, 16.05), <0.001	8.34 (3.72, 18.70), <0.001	8.34 (3.72, 18.70), <0.001
T3	1.95 (1.41, 2.70), **<0.001**	2.11 (1.43, 3.12), **0.005**	1.93 (1.28, 2.92), **0.020**	5.70 (2.68, 12.12), **<0.001**	6.10 (2.72, 13.68), **<0.001**	6.10 (2.72, 13.68), **<0.001**
*p* for trend	**<0.001**	**0.003**	**0.015**	**<0.001**	**<0.001**	**<0.001**

The tertile cutoff values of the DII are −0.12 and 1.79 in the NHANES cohort. The tertile cut-off values of the DII are −0.09 and 1.90 in the Chinese cohort. In the NHANES cohort, Model 1, non-adjusted model. Model 2, adjusted for age, gender, ethnicity, marital status, education level, PIR, smoking status, and PA level. Model 3, adjusted for age, gender, ethnicity, marital status, education level, PIR, smoking status, PA level, DM, hypertension, and stroke history. In the Chinese cohort, Model 1, non-adjusted model. Model 2, adjusted for age, gender, marital status, education level, smoking status, and PA level. Model 3, adjusted for age, gender, marital status, education level, smoking status, PA level, DM, hypertension, and stroke history. NHANES, National Health and Nutrition Examination Survey; DII, dietary inflammatory index; SO, sarcopenic obesity; PIR, family poverty-to-income ratio; PA, physical activity; DM, diabetes mellitus; OR, odds ratio; CI, confidence interval; T, tertile.

**Figure 3:**
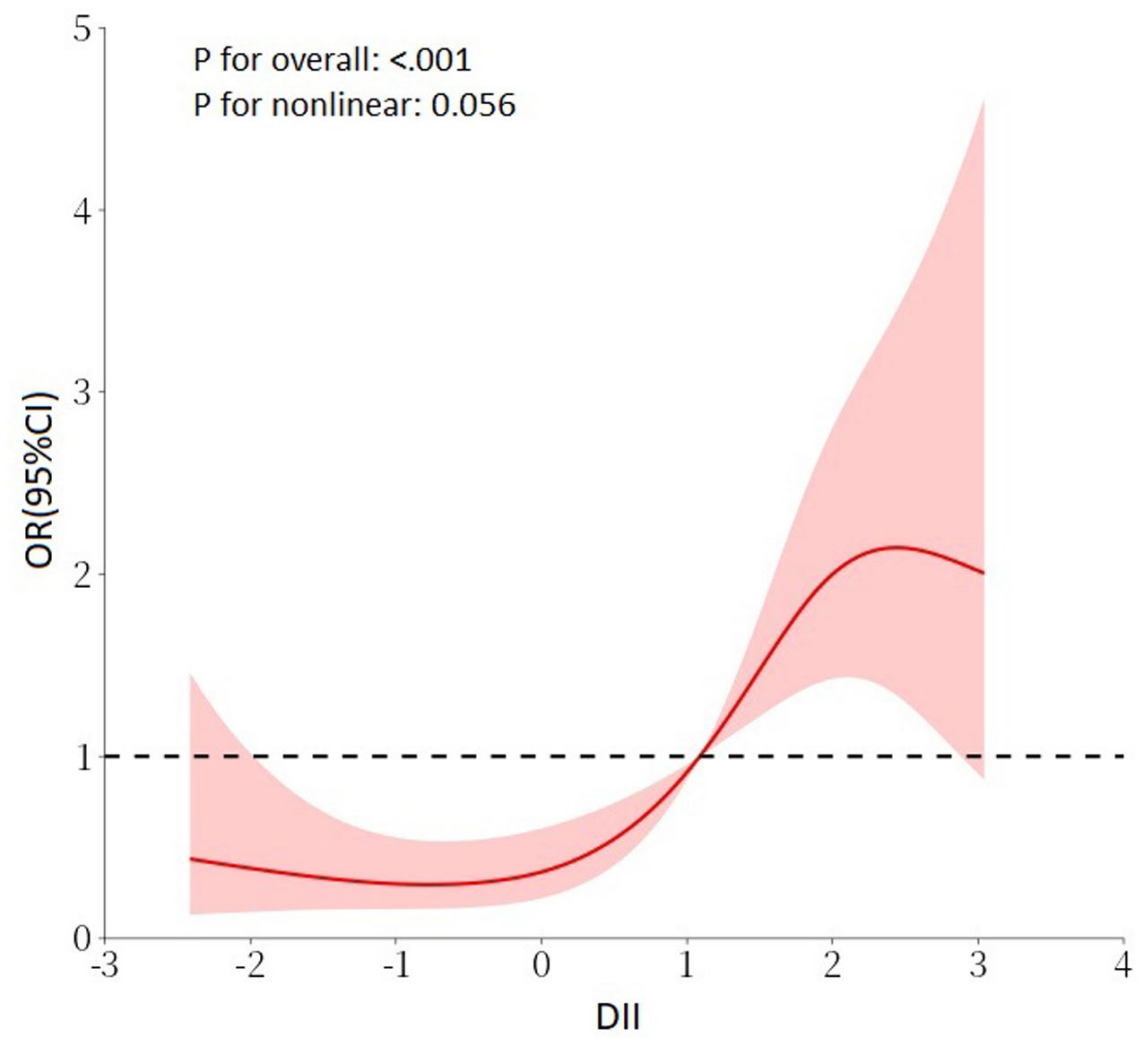
RCS regression analysis for DII and SO in the Chinese cohort. Adjusted for age, gender, marital status, education level, smoking status, PA level, DM, hypertension, and stroke history. RCS, restricted cubic spline; DII, dietary inflammatory index; SO, sarcopenic obesity; PA, physical activity; DM, diabetes mellitus.

In the Chinese cohort, we explored the relationship between DII and SO in the Chinese population cohort. The findings demonstrated significant correlations between DII and SO, which were evident in both unadjusted and multivariable-adjusted models (Model 3, OR _continuous_ = 1.59, 95% CI = 1.30, 1.94, *p* < 0.001; OR _tertile3vs1_ = 6.10, 95% CI = 2.72, 13.68, *p* for trend <0.001; Table 4). In the RCS regression analysis (Figure 3), the results showed that the association between DII and the risk of SO was monotonically increasing. All findings further emphasized the robustness and reliability of the observed trend: as DII scores increase, the risk of SO correspondingly rises.

### Mediating role of inflammatory/nutritional indicators

**Figure 4:**
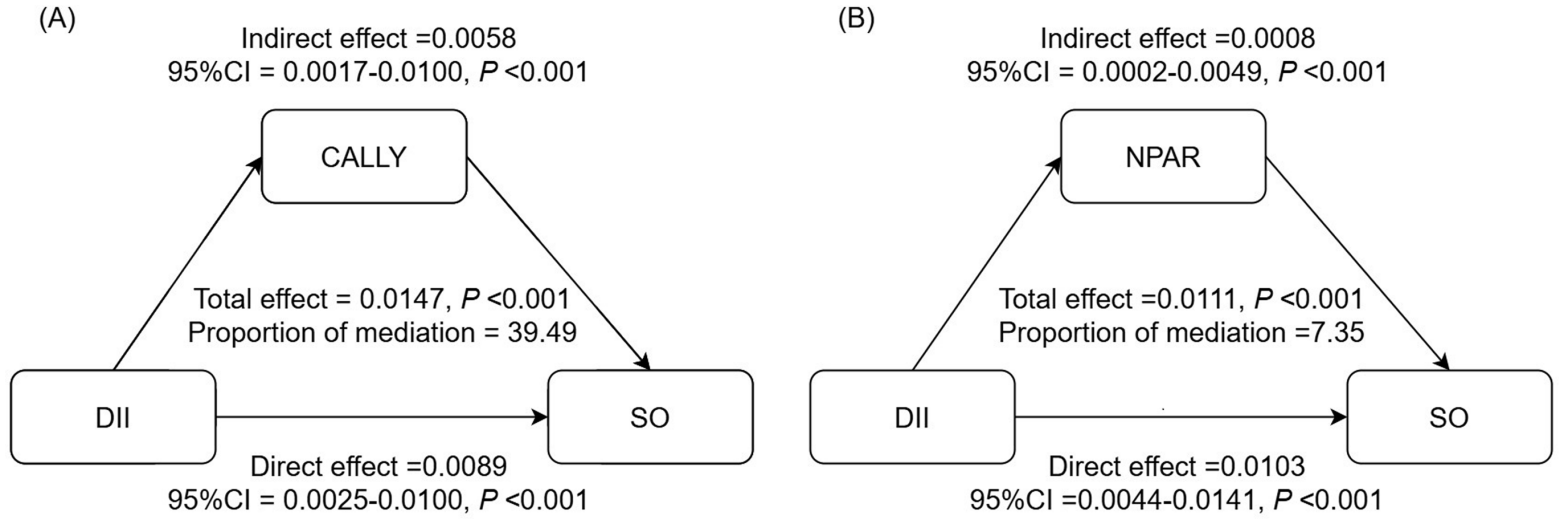
Analysis of the mediation by inflammation-related indicators of the association between DII and SO. **(A)** CALLY, **(B)** NPAR. Adjusted for adjusted for age, gender, race, marital status, education level, PIR, smoking status, PA level, DM, hypertension, and stroke history. CALLY, C-reactive protein-albumin-lymphocyte; NPAR, neutrophil-percentage-to-albumin ratio; DII, dietary inflammatory index; SO, sarcopenic obesity; CI, confidence interval.

In the NHANES cohort, the associations of inflammatory/nutritional indicators with DII and SO in both unadjusted and multivariable-adjusted models are demonstrated in Table 5. After full adjustment, DII maintained a positive association with NPAR (*β* = 0.09, 95% CI = 0.02, 0.17, *p* = 0.036, Table 5) and a negative association with CALLY (*β* = −94.67, 95% CI = −179.64, −9.70, *p* = 0.049, Table 5). Results from logistic regression analysis displayed that NPAR was positively associated with SO, while CALLY was negatively associated with SO (Table 5). Then, we explored the mediation effect of inflammatory/nutritional indicators on the association between DII and SO. The mediation analysis results indicated that CALLY mediated 39.49% of the association between DII and SO, while NPAR mediated 7.35% (Figure 4).

**Table 5:** Associations of inflammation-related indicators with DII and SO in the NHANES cohort.

Mediators	DII to mediator		Mediator to SO	
	*β* (95% CI)	*p*-value	OR (95% CI)	*p*-value
CALLY				
Model 1	−138.22 (−226.86, −49.57)	**0.005**	0.99 (0.99, 0.99)	**<0.001**
Model 2	−94.51 (−176.78, −12.25)	**0.048**	0.99 (0.99, 0.99)	**<0.001**
Model 3	−94.67 (−179.64, −9.70)	**0.049**	0.99 (0.99, 0.99)	**0.002**
NPAR				
Model 1	0.15 (0.08, 0.22)	**<0.001**	1.20 (1.11, 1.30)	**<0.001**
Model 2	0.10 (0.03, 0.17)	**0.019**	1.17 (1.07, 1.28)	**0.007**
Model 3	0.09 (0.02, 0.17)	**0.036**	1.15 (1.05, 1.26)	**0.021**

Model 1, non-adjusted model. Model 2, adjusted for age, gender, ethnicity, marital status, education level, PIR, smoking status, and PA level. Model 3, adjusted for age, gender, ethnicity, marital status, education level, PIR, smoking status, PA level, DM, hypertension, and stroke history. In the Chinese cohort, Model 1, non-adjusted model. Model 2, adjusted for age, gender, marital status, education level, smoking status, and PA level. Model 3, adjusted for age, gender, marital status, education level, smoking status, PA level, DM, hypertension, and stroke history. NHANES, National Health and Nutrition Examination Survey; DII, dietary inflammatory index; SO, sarcopenic obesity; CALLY, C-reactive protein-albumin-lymphocyte; NPAR, neutrophil-percentage-to-albumin ratio; PIR, family poverty-to-income ratio; PA, physical activity; DM, diabetes mellitus; OR, odds ratio; CI, confidence interval.

## Discussion

This study is the first to explore the association between DII and SO in U.S. and Chinese populations. The findings revealed a clear positive association between elevated DII and an increased risk of SO within the cohort. These associations remained significant even in the fully adjusted model. We further explored the potential mediating effect of inflammation/nutritional indicators within these associations and found that the associations appear to be partially mediated by inflammation/nutritional pathways.

### Chronic inflammation and SO

Chronic low-grade inflammation is considered a pivotal factor in the onset and progression of both sarcopenia and obesity (6, 37–39). Expanding adipose depots can induce an imbalanced production of various adipokines and promote the infiltration of macrophages and other immune cells (2, 3). These activities lead to the release of a diverse array of pro-inflammatory cytokines and chemokines, resulting in the initiation and sustenance of a chronic low-grade inflammation state (2, 3, 40). Inflamed AT milieu can induce insulin resistance and fat infiltration in skeletal muscle (41–43). Intramuscular lipids induce local inflammation, exacerbating in turn AT inflammation. Moreover, muscle progenitor cells may differentiate into an adipocyte-like phenotype as a result of paracrine signaling from cytokines in AT (5–7). This differentiation results in increased fatty infiltration and decreased muscular regeneration, thereby perpetuating a vicious cycle (5). The interaction between fatty infiltration and muscle loss could potentially initiate and exacerbate SO (7–9).

### Diet and inflammation

Diet is one of the most significant modifiable factors in influencing inflammation (12). Several studies revealed that diet plays a significant role in maintaining the homeostasis of the gut microenvironment (44, 45). It achieves this by regulating the release of gut hormones, influencing the composition and function of gut microbiota (GM), and affecting the integrity of the gut barrier (44, 45). Dietary components directly influence the composition and activity of the GM, which, in turn, produces metabolites that can modulate the inflammatory state and immune responses. For instance, short-chain fatty acids produced from the fermentation of dietary fiber can enhance gut barrier function and regulate inflammatory pathways, while saturated fats can promote the growth of pro-inflammatory bacteria and increase endotoxin levels, leading to systemic inflammation (45). Moreover, dietary patterns could shape and alter the GM and, consequently, influence the inflammatory state (13, 14). Excessive consumption of fat, such as in a high-fat or Western-style diet, can lead to dysbiosis, dysfunction of the gut barrier, increased intestinal permeability, and the release of toxic bacterial metabolites into the circulation, thereby strongly contributing to the development of low-grade systemic inflammation (14). Furthermore, increased consumption of ultra-processed foods could modulate low-grade inflammation (13). Growing evidence suggests that GM is integral to the pathophysiology of fatty and musculoskeletal disorders through multiple pathways, including chronic inflammation and metabolic imbalance (46–48). Therefore, it is necessary to explore the correlation between dietary inflammatory potential and SO to develop dietary treatment strategies for managing OAO and SO.

### Effects of dietary inflammatory potential on SO

The DII is a scoring tool designed to assess the inflammatory potential of diets (21). A lower DII score reflects a more anti-inflammatory diet, whereas a higher DII score indicates a more pro-inflammatory diet. Recently, the implications of dietary inflammatory potential for obesity and sarcopenia have garnered increasing attention (16, 22, 23). Many population-based studies have explored the correlation between the DII score and these conditions, highlighting the strong connection between dietary inflammation and the risk of obesity and sarcopenia (16, 23–29). However, there is limited research exploring the association between DII and SO. In the present study, we found a persistent positive association between elevated DII scores and the risk of SO among U.S. and Chinese adults using datasets from NHANES and Tongde Hospital of Zhejiang Province. This finding is inconsistent with the results from the Korean older adult population (17). This discrepancy may be attributed to differences in the study populations: the Korean cohort focused on elderly individuals aged 70–84 years, whereas our study included a broader age range. SO initially refers to a geriatric disorder characterized by the progressive loss of skeletal muscle mass and/or function, combined with excessive fat accumulation (9). Due to the age of the elderly participants in the Korean cohort, age may play a more significant role in chronic inflammation compared to the DII. However, changes in lifestyle, including poor dietary habits, low dietary quality, and SBs, have significantly contributed to a trend of younger individuals being affected by pre-SO and SO (11). Hence, we explored the relationship among individuals over the age of 20 and found a strong correlation between DII and SO.

### The mediation effect of CALLY and NPAR on DII-SO

Both chronic low-grade inflammation and malnutrition are critical risk factors for SO (8, 9, 49). Therefore, recognizing the importance of inflammatory and nutritional status in the development, prevention, and treatment of SO is imperative. Albumin, primarily produced by the liver, serves as an indicator of nutritional status. It plays a vital role in maintaining blood volume balance and transporting nutrients. Although the exact relationship between albumin and SO remains unclear, several studies have demonstrated an inverse relationship between serum albumin levels and the occurrence of sarcopenia (50, 51). CBC parameters, including neutrophils and lymphocytes, are traditional biomarkers used to evaluate immune function. Neutrophils are the first immune cells to infiltrate adipose tissue (52, 53). Once activated, they release inflammatory factors that recruit macrophages and other immune cells (52, 53). These recruited cells maintain the inflammatory state by producing cytokines and chemokines, which can spread to different parts of the body and potentially lead to a systemic inflammatory condition (52–54). Lymphocytes are closely associated with muscle satellite cells (MUSCs), which are vital for skeletal muscle regeneration (55, 56). CRP is another widely used clinical indicator that reflects inflammation levels and is related to aging (57). Two population-based studies demonstrated that a high level of CRP was independently associated with SO (57, 58). Considering the significant role of inflammatory and nutritional status in fatty infiltration and muscle regeneration, we hypothesized that the CALLY index and NPAR could predict the risk of SO and mediate the relationship between the dietary inflammatory index DII and SO.

The CALLY index, which combines albumin, lymphocyte count, and CRP levels, is an improved scoring system that reflects the immune, inflammatory, and nutritional status of individuals. It was developed as a prognostic factor for patients suffering from various cancers (59–63). Recent research revealed that elevated CALLY levels are independently related to a decreased risk of sarcopenia in both community residents from the NHANES cohort in the U.S. and hospitalized patients from the Kunshan Hospital cohort in China (33). In our study, significant correlations of CALLY with DII and SO were demonstrated in both crude and multivariable-adjusted models. The NPAR, which combines neutrophil percentage and albumin, is a prognostic factor for patients suffering from various chronic non-communicable diseases such as heart failure, chronic obstructive pulmonary disease, metabolic syndrome, and non-alcoholic fatty liver disease (34, 64–67). Our results demonstrated positive associations of NPAR with DII and SO in both crude and multivariable-adjusted models. After exploring the associations of inflammatory/nutritional indicators with DII and SO, a mediation analysis was conducted, emphasizing the significant roles of CALLY and NPAR in linking DII with SO. This suggests an underlying inflammatory/nutritional mechanism in these associations.

### Strengths and limitations

The primary strength of the present study is its foundation on two diverse population cohorts: participants from the NHANES cohort in the U.S. and patients from Tongde Hospital of Zhejiang Province in China. The comprehensive and representative characteristics of the NHANES database significantly enhance the generalizability of our findings. Additionally, the study demonstrated the broad applicability of the DII score through its analysis of the Chinese cohort from Tongde Hospital, facilitating a detailed examination of the relationship between DII and SO. However, the study has several limitations. First, inherent differences in baseline characteristics between clinic patients and community survey participants introduce confounding effects that may influence the results. Second, the cross-sectional study design may limit the ability to draw definitive conclusions about the causal relationship between DII and SO outcomes. Third, the sample size of the clinical cohort was relatively small. Therefore, further external validation is necessary to confirm the association between DII and SO, particularly among hospitalized patients. Despite these limitations, our findings reveal a close connection between DII and SO, which could potentially guide future practical applications.

## Conclusion

An elevated DII score is positively associated with the risk of SO in adults. This association appears to be partially mediated by inflammatory/nutritional pathways, indicating that the DII score may serve as a valuable indicator for identifying individuals at risk of SO. Adults may reduce their risk of SO by decreasing the inflammatory potential in their daily diets.

## Data Availability

The original contributions presented in the study are included in the article/Supplementary material, further inquiries can be directed to the corresponding author.

## Footnotes

^1^ https://www.cdc.gov/nchs/nhanes/about_nhanes.htm

^2^ http://www.R-project.org

^3^ www.medsta.cn/software

^4^ www.empowerstats.net

## References

[1] Blüher M. Obesity: global epidemiology and pathogenesis. Nat Rev Endocrinol. (2019) 15:288–98. doi: 10.1038/s41574-019-0176-8, PMID: 30814686

[2] Suren Garg S Kushwaha K Dubey R Gupta J. Association between obesity, inflammation and insulin resistance: insights into signaling pathways and therapeutic interventions. Diabetes Res Clin Pract. (2023) 200:110691. doi: 10.1016/j.diabres.2023.110691, PMID: 37150407

[3] Uranga RM Keller JN. The complex interactions between obesity, metabolism and the brain. Front Neurosci. (2019) 13:513. doi: 10.3389/fnins.2019.00513, PMID: 31178685 PMC6542999

[4] Zhang X Li H He M Wang J Wu Y Li Y. Immune system and sarcopenia: presented relationship and future perspective. Exp Gerontol. (2022) 164:111823. doi: 10.1016/j.exger.2022.111823, PMID: 35504482

[5] Li CW Yu K Shyh-Chang N Jiang Z Liu T Ma S. Pathogenesis of sarcopenia and the relationship with fat mass: descriptive review. J Cachexia Sarcopenia Muscle. (2022) 13:781–94. doi: 10.1002/jcsm.12901, PMID: 35106971 PMC8977978

[6] Jimenez-Gutierrez GE Martínez-Gómez LE Martínez-Armenta C Pineda C Martínez-Nava GA Lopez-Reyes A. Molecular mechanisms of inflammation in sarcopenia: diagnosis and therapeutic update. Cells. (2022) 11:2359. doi: 10.3390/cells11152359, PMID: 35954203 PMC9367570

[7] Tardif N Salles J Guillet C Tordjman J Reggio S Landrier JF. Muscle ectopic fat deposition contributes to anabolic resistance in obese sarcopenic old rats through eIF2α activation. Aging Cell. (2014) 13:1001–11. doi: 10.1111/acel.12263, PMID: 25139155 PMC4326920

[8] Kalinkovich A Livshits G. Sarcopenic obesity or obese sarcopenia: a cross talk between age-associated adipose tissue and skeletal muscle inflammation as a main mechanism of the pathogenesis. Ageing Res Rev. (2017) 35:200–21. doi: 10.1016/j.arr.2016.09.008 27702700

[9] Donini LM Busetto L Bischoff SC Cederholm T Ballesteros-Pomar MD Batsis JA. Definition and diagnostic criteria for sarcopenic obesity: ESPEN and EASO consensus statement. Clin Nutr. (2022) 41:990–1000. doi: 10.1016/j.clnu.2021.11.014, PMID: 35227529

[10] Benz E Pinel A Guillet C Capel F Pereira B De Antonio M. Sarcopenia and sarcopenic obesity and mortality among older people. JAMA Netw Open. (2024) 7:e243604. doi: 10.1001/jamanetworkopen.2024.3604, PMID: 38526491 PMC10964118

[11] Ji T Li Y Ma L. Sarcopenic obesity: an emerging public health problem. Aging Dis. (2022) 13:379–88. doi: 10.14336/AD.2021.1006, PMID: 35371597 PMC8947824

[12] Galland L. Diet and inflammation. Nutr Clin Pract. (2010) 25:634–40. doi: 10.1177/0884533610385703 21139128

[13] Tristan Asensi M Napoletano A Sofi F Dinu M. Low-grade inflammation and ultra-processed foods consumption: a review. Nutrients. (2023) 15:1546. doi: 10.3390/nu15061546, PMID: 36986276 PMC10058108

[14] Malesza IJ Malesza M Walkowiak J Mussin N Walkowiak D Aringazina R. High-fat, Western-style diet, systemic inflammation, and gut microbiota: a narrative review. Cells. (2021) 10:3164. doi: 10.3390/cells10113164, PMID: 34831387 PMC8619527

[15] Hariharan R Odjidja EN Scott D Shivappa N Hébert JR Hodge A. The dietary inflammatory index, obesity, type 2 diabetes, and cardiovascular risk factors and diseases. Obes Rev. (2022) 23:e13349. doi: 10.1111/obr.13349, PMID: 34708499

[16] Wang YB Shivappa N Hébert JR Page AJ Gill TK Melaku YA. Association between dietary inflammatory index, dietary patterns, plant-based dietary index and the risk of obesity. Nutrients. (2021) 13:1536. doi: 10.3390/nu13051536, PMID: 34063221 PMC8147427

[17] Jung S Lee Y Kim K Park S. Association of the dietary inflammatory index with sarcopenic obesity and frailty in older adults. BMC Geriatr. (2024) 24:654. doi: 10.1186/s12877-024-05239-z, PMID: 39097690 PMC11297761

[18] Zhou N Xie ZP Liu Q Xu Y Dai SC Lu J. The dietary inflammatory index and its association with the prevalence of hypertension: a cross-sectional study. Front Immunol. (2023) 13:1097228. doi: 10.3389/fimmu.2022.1097228, PMID: 36741368 PMC9893776

[19] Liu Z Liu H Deng Q Sun C He W Zheng W. Association between dietary inflammatory index and heart failure: results from NHANES (1999-2018). Front Cardiovasc Med. (2021) 8:702489. doi: 10.3389/fcvm.2021.702489, PMID: 34307508 PMC8292138

[20] Mao Y Weng J Xie Q Wu L Xuan Y Zhang J. Association between dietary inflammatory index and stroke in the US population: evidence from NHANES 1999-2018. BMC Public Health. (2024) 24:50. doi: 10.1186/s12889-023-17556-w, PMID: 38166986 PMC10763382

[21] Shivappa N Steck SE Hurley TG Hussey JR Hébert JR. Designing and developing a literature-derived, population-based dietary inflammatory index. Public Health Nutr. (2014) 17:1689–96. doi: 10.1017/S1368980013002115, PMID: 23941862 PMC3925198

[22] Bloom I Shand C Cooper C Robinson S Baird J. Diet quality and sarcopenia in older adults: a systematic review. Nutrients. (2018) 10:308. doi: 10.3390/nu10030308, PMID: 29510572 PMC5872726

[23] Shirinyfard Pilehrood K Askari G Sharifi M Kargarfard M Saraf-Bank S. Elevated risk of possible sarcopenia and weak muscle strength with higher dietary inflammatory index in Iranian breast cancer survivors: a cross-sectional study. BMC Nutr. (2025) 11:5. doi: 10.1186/s40795-025-00992-9, PMID: 39789664 PMC11721246

[24] Pu R Man Q Song S Jia S Liu Z Zhang X. The dietary inflammatory index and sarcopenia in older adults in four Chinese provinces: a cross-sectional study. Nutrients. (2025) 17:478. doi: 10.3390/nu17030478, PMID: 39940334 PMC11820900

[25] Zheng G Xia H Lai Z Shi H Zhang J Wang C. Dietary inflammatory index and dietary diversity score associated with sarcopenia and its components: findings from a nationwide cross-sectional study. Nutrients. (2024) 16:1038. doi: 10.3390/nu16071038, PMID: 38613070 PMC11013103

[26] Bian D Liu X Wang C Jiang Y Gu Y Zhong J. Association between dietary inflammatory index and sarcopenia in Crohn’s disease patients. Nutrients. (2022) 14:901. doi: 10.3390/nu14040901, PMID: 35215553 PMC8878789

[27] Son BK Akishita M Yamanaka T Toyoshima K Tanaka T Suthutvoravut U. Association between inflammatory potential of the diet and sarcopenia/its components in community-dwelling older Japanese men. Arch Gerontol Geriatr. (2021) 97:104481. doi: 10.1016/j.archger.2021.104481, PMID: 34298260

[28] Bagheri A Soltani S Hashemi R Heshmat R Motlagh AD Esmaillzadeh A. Inflammatory potential of the diet and risk of sarcopenia and its components. Nutr J. (2020) 19:129. doi: 10.1186/s12937-020-00649-2, PMID: 33248463 PMC7700703

[29] Granic A Sayer AA Robinson SM. Dietary patterns, skeletal muscle health, and sarcopenia in older adults. Nutrients. (2019) 11:745. doi: 10.3390/nu11040745, PMID: 30935012 PMC6521630

[30] Zheng Y Liu W Zhu X Xu M Lin B Bai Y. Associations of dietary inflammation index and composite dietary antioxidant index with preserved ratio impaired spirometry in US adults and the mediating roles of triglyceride-glucose index: NHANES 2007-2012. Redox Biol. (2024) 76:103334. doi: 10.1016/j.redox.2024.103334, PMID: 39217849 PMC11402638

[31] Yang J Liu C Zhao S Wang L Wu G Zhao Z. The association between the triglyceride-glucose index and sarcopenia: data from the NHANES 2011-2018. Lipids Health Dis. (2024) 23:219. doi: 10.1186/s12944-024-02201-1, PMID: 39030624 PMC11264742

[32] Chen LK Woo J Assantachai P Auyeung TW Chou MY Iijima K. Asian Working Group for Sarcopenia: 2019 consensus update on sarcopenia diagnosis and treatment. J Am Med Dir Assoc. (2020) 21:300–307.e2. doi: 10.1016/j.jamda.2019.12.012, PMID: 32033882

[33] Li Y Wei Q Ke X Xu Y Xu B Zhang K. Higher CALLY index levels indicate lower sarcopenia risk among middle-aged and elderly community residents as well as hospitalized patients. Sci Rep. (2024) 14:24591. doi: 10.1038/s41598-024-75164-z, PMID: 39426987 PMC11490578

[34] Liu CF Chien LW. Predictive role of neutrophil-percentage-to-albumin ratio (NPAR) in nonalcoholic fatty liver disease and advanced liver fibrosis in nondiabetic US adults: evidence from NHANES 2017-2018. Nutrients. (2023) 15:1892. doi: 10.3390/nu15081892, PMID: 37111111 PMC10141547

[35] MacGregor KA Gallagher IJ Moran CN. Relationship between insulin sensitivity and menstrual cycle is modified by BMI, fitness, and physical activity in NHANES. J Clin Endocrinol Metab. (2021) 106:2979–90. doi: 10.1210/clinem/dgab415, PMID: 34111293 PMC8475204

[36] Craig CL Marshall AL Sjöström M Bauman AE Booth ML Ainsworth BE. International physical activity questionnaire: 12-country reliability and validity. Med Sci Sports Exerc. (2003) 35:1381–95. doi: 10.1249/01.MSS.0000078924.61453, PMID: 12900694

[37] Sayer AA Cooper R Arai H Cawthon PM Ntsama Essomba MJ Fielding RA. Sarcopenia. Nat Rev Dis Primers. (2024) 10:68. doi: 10.1038/s41572-024-00550-w 39300120

[38] Schleh MW Caslin HL Garcia JN Mashayekhi M Srivastava G Bradley AB. Metaflammation in obesity and its therapeutic targeting. Sci Transl Med. (2023) 15:eadf9382. doi: 10.1126/scitranslmed.adf9382, PMID: 37992150 PMC10847980

[39] Kawai T Autieri MV Scalia R. Adipose tissue inflammation and metabolic dysfunction in obesity. Am J Physiol Cell Physiol. (2021) 320:C375–91. doi: 10.1152/ajpcell.00379.2020, PMID: 33356944 PMC8294624

[40] Guzik TJ Skiba DS Touyz RM Harrison DG. The role of infiltrating immune cells in dysfunctional adipose tissue. Cardiovasc Res. (2017) 113:1009–23. doi: 10.1093/cvr/cvx108, PMID: 28838042 PMC5852626

[41] Wu Z Yang J Zhu Y Li J Xu K Li Y. Causal associations of inflammatory cytokines with osteosarcopenia: insights from mendelian randomization and single cell analysis. Mediat Inflamm. (2025) 2025:6005225. doi: 10.1155/mi/6005225, PMID: 40224485 PMC11986192

[42] Gouda M Lv JM Huang Z Chen JC He Y Li X. Bioprobe-RNA-seq-microRaman system for deep tracking of the live single-cell metabolic pathway chemometrics. Biosens Bioelectron. (2024) 261:116504. doi: 10.1016/j.bios.2024.116504, PMID: 38896978

[43] Xu Z You W Chen W Zhou Y Nong Q Valencak TG. Single-cell RNA sequencing and lipidomics reveal cell and lipid dynamics of fat infiltration in skeletal muscle. J Cachexia Sarcopenia Muscle. (2021) 12:109–29. doi: 10.1002/jcsm.12643, PMID: 33244879 PMC7890272

[44] Beam A Clinger E Hao L. Effect of diet and dietary components on the composition of the gut microbiota. Nutrients. (2021) 13:2795. doi: 10.3390/nu13082795, PMID: 34444955 PMC8398149

[45] Randeni N Bordiga M Xu B. A comprehensive review of the triangular relationship among diet-gut microbiota-inflammation. Int J Mol Sci. (2024) 25:9366. doi: 10.3390/ijms25179366, PMID: 39273314 PMC11394685

[46] Mai X Yang S Chen Q Chen K. Gut microbial composition is altered in sarcopenia: a systematic review and meta-analysis of clinical studies. PLoS One. (2024) 19:e0308360. doi: 10.1371/journal.pone.0308360, PMID: 39106230 PMC11302912

[47] Gill PA Inniss S Kumagai T Rahman FZ Smith AM. The role of diet and gut microbiota in regulating gastrointestinal and inflammatory disease. Front Immunol. (2022) 13:866059. doi: 10.3389/fimmu.2022.866059, PMID: 35450067 PMC9016115

[48] Asadi A Shadab Mehr N Mohamadi MH Shokri F Heidary M Sadeghifard N. Obesity and gut-microbiota-brain axis: a narrative review. J Clin Lab Anal. (2022) 36:e24420. doi: 10.1002/jcla.24420, PMID: 35421277 PMC9102524

[49] Demirdağ F Kolbaşı EN Yildiz GK. The association between sarcopenic obesity and malnutrition in community-dwelling older adults. Age Ageing. (2025) 54:afaf040. doi: 10.1093/ageing/afaf040, PMID: 40036320

[50] Picca A Coelho-Junior HJ Calvani R Marzetti E Vetrano DL. Biomarkers shared by frailty and sarcopenia in older adults: a systematic review and meta-analysis. Ageing Res Rev. (2022) 73:101530. doi: 10.1016/j.arr.2021.101530, PMID: 34839041

[51] Silva-Fhon JR Rojas-Huayta VM Aparco-Balboa JP Céspedes-Panduro B Partezani-Rodrigues RA. Sarcopenia and blood albumin: a systematic review with meta-analysis. Biomedica. (2021) 41:590–603. doi: 10.7705/biomedica.5765, PMID: 34559500 PMC8527986

[52] Uribe-Querol E Rosales C. Neutrophils actively contribute to obesity-associated inflammation and pathological complications. Cells. (2022) 11:1883. doi: 10.3390/cells11121883, PMID: 35741012 PMC9221045

[53] Altamura S Lombardi F Palumbo P Cinque B Ferri C Del Pinto R. The evolving role of neutrophils and neutrophil extracellular traps (NETs) in obesity and related diseases: recent insights and advances. Int J Mol Sci. (2024) 25:136 33. doi: 10.3390/ijms252413633, PMID: 39769394 PMC11727698

[54] Liew PX Kubes P. The neutrophil's role during health and disease. Physiol Rev. (2019) 99:1223–48. doi: 10.1152/physrev.00012.2018, PMID: 30758246

[55] Brack AS Rando TA. Tissue-specific stem cells: lessons from the skeletal muscle satellite cell. Cell Stem Cell. (2012) 10:504–14. doi: 10.1016/j.stem.2012.04.001, PMID: 22560074 PMC3348769

[56] Henrot P Blervaque L Dupin I Zysman M Esteves P Gouzi F. Cellular interplay in skeletal muscle regeneration and wasting: insights from animal models. J Cachexia Sarcopenia Muscle. (2023) 14:745–57. doi: 10.1002/jcsm.13103, PMID: 36811134 PMC10067506

[57] Park CH Do JG Lee YT Yoon KJ. Sarcopenic obesity associated with high-sensitivity C-reactive protein in age and sex comparison: a two-center study in South Korea. BMJ Open. (2018) 8:e021232. doi: 10.1136/bmjopen-2017-021232, PMID: 30232104 PMC6150137

[58] Dutra MT Avelar BP Souza VC Bottaro M Oliveira RJ Nóbrega OT. Relationship between sarcopenic obesity-related phenotypes and inflammatory markers in post*menopausal women*. Clin Physiol Funct Imaging. (2017) 37:205–10. doi: 10.1111/cpf.12287, PMID: 26373437

[59] Müller L Hahn F Mähringer-Kunz A Stoehr F Gairing SJ Michel M. Immunonutritive scoring for patients with hepatocellular carcinoma undergoing transarterial chemoembolization: evaluation of the CALLY index. Cancers (Basel). (2021) 13:5018. doi: 10.3390/cancers13195018, PMID: 34638502 PMC8508385

[60] Tsai YT Ko CA Chen HC Hsu CM Lai CH Lee YC. Prognostic value of CRP-albumin-lymphocyte (CALLY) index in patients undergoing surgery for oral cavity cancer. J Cancer. (2022) 13:3000–12. doi: 10.7150/jca.74930, PMID: 36046647 PMC9414026

[61] Furukawa K Tsunematsu M Tanji Y Ishizaki S Akaoka M Haruki K. Impact of C-reactive protein-albumin-lymphocyte (CALLY) index on prognosis after hepatectomy for colorectal liver metastasis. Surg Oncol. (2023) 47:101911. doi: 10.1016/j.suronc.2023.101911, PMID: 36773544

[62] Zhuang J Wang S Wang Y Wu Y Hu R. Prognostic value of CRP-albumin-lymphocyte (CALLY) index in patients undergoing surgery for breast cancer. Int J Gen Med. (2024) 17:997–1005. doi: 10.2147/IJGM.S447201, PMID: 38505146 PMC10949993

[63] Fukushima N Masuda T Tsuboi K Takahashi K Yuda M Fujisaki M. Prognostic significance of the preoperative C-reactive protein-albumin-lymphocyte (CALLY) index on outcomes after gastrectomy for gastric cancer. Surg Today. (2024) 54:943–52. doi: 10.1007/s00595-024-02813-1, PMID: 38491233

[64] Wu CC Wu CH Lee CH Cheng CI. Association between neutrophil percentage-to-albumin ratio (NPAR), neutrophil-to-lymphocyte ratio (NLR), platelet-to-lymphocyte ratio (PLR) and long-term mortality in community-dwelling adults with heart failure: evidence from US NHANES 2005-2016. BMC Cardiovasc Disord. (2023) 23:312. doi: 10.1186/s12872-023-03316-6, PMID: 37344786 PMC10286403

[65] Lan CC Su WL Yang MC Chen SY Wu YK. Predictive role of neutrophil-percentage-to-albumin, neutrophil-to-lymphocyte and eosinophil-to-lymphocyte ratios for mortality in patients with COPD: evidence from NHANES 2011-2018. Respirology. (2023) 28:1136–46. doi: 10.1111/resp.14589, PMID: 37655985

[66] Ji W Li H Qi Y Zhou W Chang Y Xu D. Association between neutrophil-percentage-to-albumin ratio (NPAR) and metabolic syndrome risk: insights from a large US population-based study. Sci Rep. (2024) 14:26646. doi: 10.1038/s41598-024-77802-y, PMID: 39496695 PMC11535182

[67] Cucoranu DC Pop M Niculescu R Kosovski IB Toganel RO Licu RA. The association of nonalcoholic fatty liver disease with neutrophil-to-lymphocyte ratio and neutrophil-percentage-to-albumin ratio. Cureus. (2023) 15:e41197. doi: 10.7759/cureus.41197, PMID: 37525801 PMC10387286

