# Supplementary figures and images for: The impact of elevated dietary inflammatory potential on sarcopenic obesity: evidence from two observational studies
Source: Front Nutr. 2025 Aug 11;12:1621199. doi: 10.3389/fnut.2025.1621199 (PMC12375489; doi:10.3389/fnut.2025.1621199)

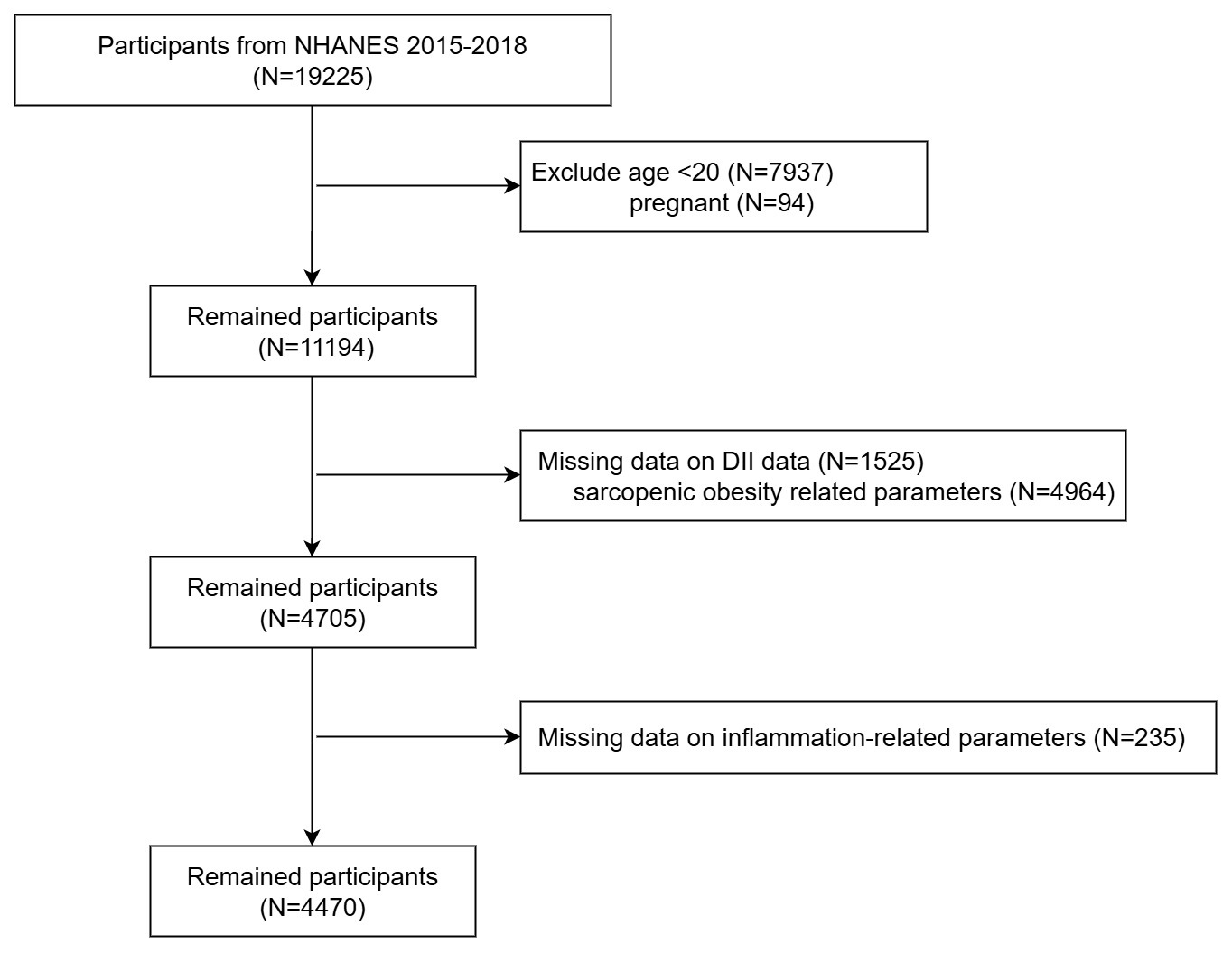

Supplement: Supplementary file 1 [file Image_1.JPEG]

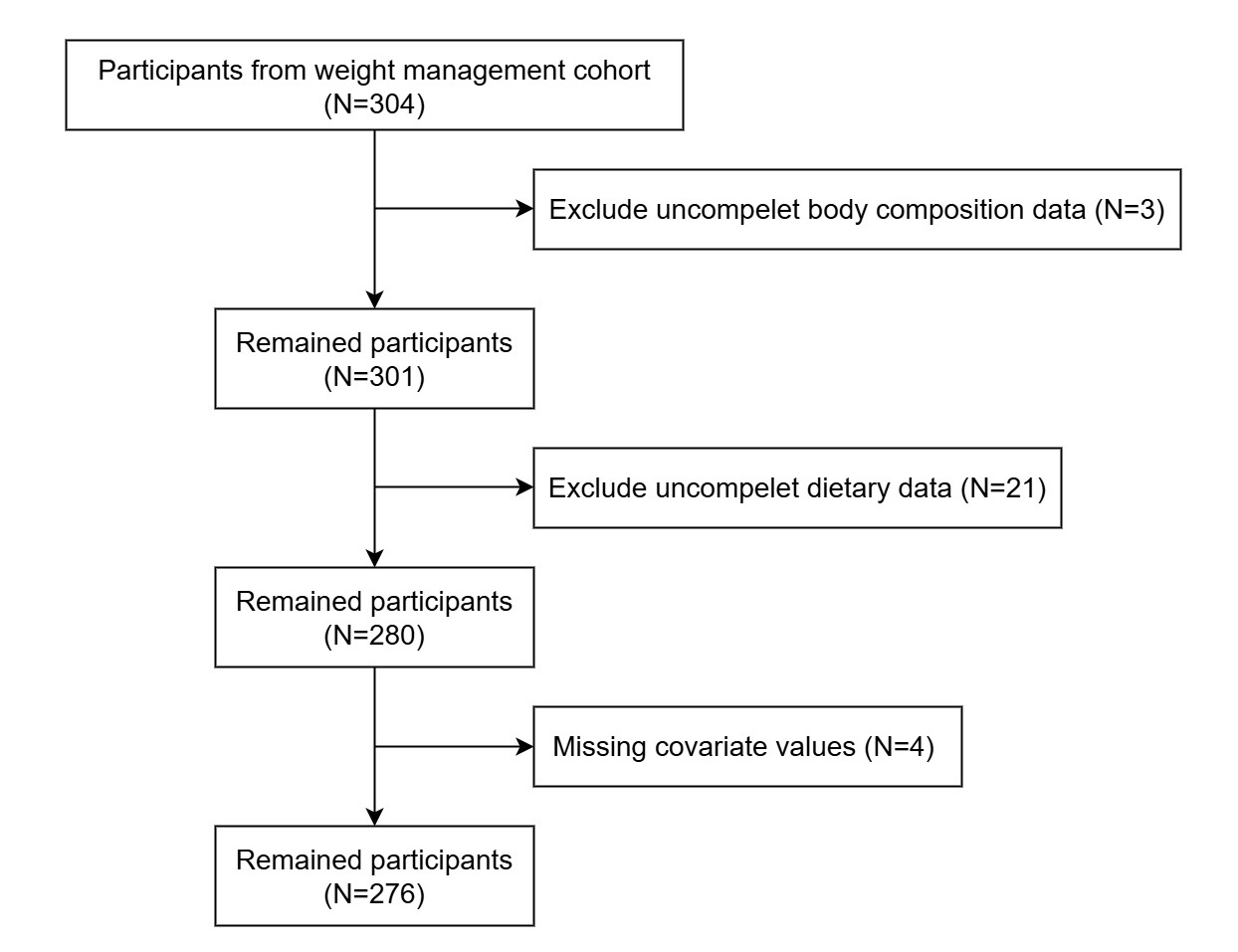

Supplement: Supplementary file 2 [file Image_2.JPEG]
