# Supplementary material for: The impact of elevated dietary inflammatory potential on sarcopenic obesity: evidence from two observational studies
Source: Front Nutr. 2025 Aug 11;12:1621199. doi: 10.3389/fnut.2025.1621199 (PMC12375489; doi:10.3389/fnut.2025.1621199)
Supplement: Supplementary file 3 [file Table_1.docx]

**Supplement Table 1.** Subgroup analysis between DII and SO in the NHANES cohort.

| Variables | OR (95% CI) | *P* | *P* for interaction |
| --- | --- | --- | --- |
| All patients | 1.19 (1.08, 1.32) | 0.012 |  |
| Gender |  |  | 0.743 |
| Male | 1.19 (1.06, 1.33) | 0.019 |  |
| Female | 1.17 (0.96, 1.43) | 0.148 |  |
| BMI |  |  | 0.170 |
| <25.0 | 1.00 (0.96, 1.05) | 0.895 |  |
| ≥25.0, <30.0 | 1.41 (1.21, 1.65) | 0.003 |  |
| ≥30.0 | 1.11 (0.98, 1.27) | 0.150 |  |
| Central obesity |  |  | 0.637 |
| Yes | 1.14 (1.00, 1.30) | 0.085 |  |
| No | 1.33 (1.11, 1.58) | 0.016 |  |
| Race/ethnicity |  |  | 0.450 |
| Mexican American | 1.13 (1.01, 1.26) | 0.283 |  |
| Other Hispanic | 1.21 (1.00, 1.48) | 0.089 |  |
| Non-Hispanic White | 1.26 (0.99, 1.60) | 0.091 |  |
| Non-Hispanic Black | 1.51 (1.10, 2.07) | 0.049 |  |
| Other Race-Including Multi-Racial | 1.16 (1.00, 1.35) | 0.085 |  |
| Education level |  |  | 0.627 |
| Less Than 9th Grade | 1.30 (1.09, 1.55) | 0.032 |  |
| 9-11th Grade | 1.09 (0.93, 1.27) | 0.310 |  |
| High School or Equivalent | 1.21 (1.07, 1.37) | 0.011 |  |
| Some College or AA degree | 1.10 (0.88, 1.38) | 0.417 |  |
| College Graduate or above | 1.35 (1.14, 1.59) | 0.006 |  |
| Marital status |  |  | 0.110 |
| Married | 1.08 (0.93, 1.26) | 0.321 |  |
| Divorced | 1.26 (0.97, 1.63) | 0.106 |  |
| Separated | 1.06 (0.73, 1.56) | 0.762 |  |
| Never married | 1.26 (1.03, 1.54) | 0.040 |  |
| Living with partner | 1.72 (1.29, 2.29) | 0.003 |  |
| Smoking status |  |  | 0.258 |
| Never | 1.31 (1.16, 1.47) | 0.002 |  |
| Former | 1.01 (0.79, 1.28) | 0.956 |  |
| Now | 1.21 (1.03, 1.42) | 0.048 |  |
| PA status |  |  | 0.514 |
| Low PA | 1.17 (0.99, 1.38) | 0.099 |  |
| High PA | 1.21 (1.07, 1.38) | 0.019 |  |
| Hypertension |  |  | 0.699 |
| Yes | 1.20 (0.95, 1.51) | 0.165 |  |
| No | 1.20 (1.10, 1.32) | 0.004 |  |
| DM |  |  | 0.810 |
| Yes | 1.13 (0.93, 1.37) | 0.242 |  |
| No | 1.18 (1.04, 1.34) | 0.030 |  |
| Stroke |  |  | 0.483 |
| Yes | 1.44 (0.28, 7.45) |  |  |
| No | 1.19 (1.07, 1.33) | 0.001 |  |

Adjusted for adjusted for age, gender, race, marital status, education level, PIR, smoking status, PA level, DM, hypertension, and stroke history. The model is not adjusted for the stratification variable. DII, dietary inflammatory index; SO, sarcopenic obesity; PIR, family poverty-to-income ratio; PA, physical activity; BMI, body mass index; WC, waist circumference; DM, diabetes mellitus; OR, odd ratio; CI, confidence interval.
